# Beliefs that explain mental health help-seeking intention among older men: a reasoned action approach

**DOI:** 10.1093/heapro/daaf170

**Published:** 2025-10-06

**Authors:** Grant Duthie, Hao Xu, Amy Morgan, Nicola Reavley

**Affiliations:** Centre for Mental Health and Community Wellbeing, Melbourne School of Population and Global Health, The University of Melbourne, 207 Bouverie Street, Carlton, Victoria 3010, Australia; School of Culture and Communication, The University of Melbourne, John Medley Building, Parkville, Victoria 3010, Australia; Centre for Mental Health and Community Wellbeing, Melbourne School of Population and Global Health, The University of Melbourne, 207 Bouverie Street, Carlton, Victoria 3010, Australia; Centre for Mental Health and Community Wellbeing, Melbourne School of Population and Global Health, The University of Melbourne, 207 Bouverie Street, Carlton, Victoria 3010, Australia

**Keywords:** older men, mental health, help-seeking, beliefs, attitudes, rural, campaigns

## Abstract

Older men seek help for mental health concerns at almost half the rate of older females. Efforts to motivate older men to seek help through health promotion messaging are an important public health initiative that can complement healthcare system changes. Guided by the reasoned action approach, this study aimed to identify the most salient beliefs that shape help-seeking intention among older men by region (i.e. city or rural area) to primarily inform campaign messaging and improve mental health help-seeking among older men. A mixed-methods enquiry was conducted across two samples of older men, comprising open-ended belief elicitation interviews (*N*_1_ = 23, *M*_1_ age = 74.91) and an online closed-question survey (*N*_2_ = 306 participants, *M*_2_ age = 75.28). Interviews identified 52 behavioural, normative, and control beliefs. Survey results showed salient beliefs influencing help-seeking intention (range *β* = 0.42 to *β* = 0.44), including encouragement from others, gaining new solutions, improved self-understanding, and being listened to and supported when seeking help, representing suitable messaging design candidates. The influence of salient beliefs on intention did not differ by region. The results suggest that health promotion efforts grounded in reasoned action approaches can support the development of tailored interventions to promote help-seeking for poor mental health. However, research is needed to evaluate how to effectively translate salient beliefs into carefully framed mental health messages, aligned with broader healthcare system-level reforms to promote help-seeking among older men.

Contribution to Health Promotion

- These findings highlight that health promotion messages should target beliefs around encouragement from others, gaining new solutions, improved self-understanding, and being listened to and supported to improve older men’s help-seeking for mental health concerns.
- While existing interventions have targeted some of these beliefs, this study identified several additional, underemphasized beliefs that may be particularly important in shaping older men’s help-seeking intention.
- Future campaigns for older men may be more effective by focusing on these newly identified belief targets.
- Campaign messaging should also match actual mental health service experiences among older men, which may prompt health system reforms.

## INTRODUCTION

Older men consistently use mental health services at lower rates than women, a trend observed across several high-income countries (Vasiliadis *et al*. 2013, Roxo *et al*. 2021). For instance, Australian males aged 65 years and older use mental health services at almost half the rate of older females (Bartholomaeus *et al*. 2023), with older men among the least likely age groups to seek professional help when needed (Mackenzie *et al*. 2012). A significant factor in this treatment gap involves men’s reluctance to seek help for poor mental health (Johnson *et al*. 2012 , Oliffe *et al*. 2020). The treatment gap may be even wider among older men in rural areas (Crnek-Georgeson *et al*. 2017). Untreated mental health concerns substantially reduce the quality of life and health outcomes of older adults while increasing their risk of cognitive decline and suicide (Newmark *et al*. 2020). Despite lower rates of mental disorders among older men (14.5%) than women (19.9%) (Australian Bureau of Statistics 2023b), male deaths by suicide are three times higher than that of women in high-income countries (Australian Institute of Health and Welfare 2023, Centers for Disease Control and Prevention 2023). Suicide rates among older men continue to increase with age (Shah *et al*. 2016), with men over the age of 85 having the highest suicide rate among all age groups (Australian Bureau of Statistics 2023a, Garnett *et al*. 2023). Older males living in rural areas are also more vulnerable to suicide than their metropolitan counterparts (Crnek-Georgeson *et al*. 2017). The higher male suicide rate may be partly due to men’s lower rate of help-seeking before suicide compared to females (Stene-Larsen and Reneflot 2017, Tang *et al*. 2021). Relative to younger cohorts, older adults experience various age-specific stressors such as grief, social isolation, fear of death, poor physical health, and decreased independence (Hannaford *et al*. 2019). The consequences of and stressors contributing to low help-seeking highlight the importance of addressing barriers preventing older men from seeking help (Mansfield *et al*. 2008).

Various factors contribute to the treatment gap among men across the lifespan. Specifically, traditional hegemonic masculine traits such as self-reliance, stoicism, and avoidance of emotions may hinder help-seeking for mental health (Addis and Mahalik 2003, Seidler *et al*. 2016, 2021, Fisher *et al*. 2024), while older men may prioritize masculine traits around independence, discouraging help-seeking (Smith *et al*. 2007). Rural men may also be deterred from seeking help from rural mythologies, reinforcing independence and hardiness (Piatkowski *et al*. 2023). Avoidance of blaming men around help-seeking is crucial, as mental healthcare services may also be inadequately responsive to masculine ideals (Whitley 2018, Rice *et al*. 2020). Men may face difficulties building rapport and disclosing mental health concerns with health professionals, together with barriers including service costs, long waitlists, and inaccessibility (Yousaf *et al*. 2015, Rice *et al*. 2020, Kwon *et al*. 2023). Additionally, men’s help-seeking may be inhibited by attitudinal and belief barriers, including stigma and doubts about treatment effectiveness, as well as limited knowledge about treatments, their benefits, when, how, and why to access services (Oliffe *et al*. 2016, Giallo *et al*. 2017, Seidler *et al*. 2019).

A multipronged approach addressing varied barriers hindering older men’s help-seeking is an important public health goal (Seidler *et al*. 2019). To address structural barriers, re-orienting mental health services to adopt more male-sensitive approaches should be prioritized, such as normalizing men’s help-seeking experiences and assisting GPs in minimizing stigma (Rice *et al*. 2020). There is strong and growing potential for formal mental health promotion interventions with older men (Bartlett *et al*. 2008, Pettigrew *et al*. 2012, Foettinger *et al*. 2022), including media-based campaigns, shown to shift men’s help-seeking attitudes, intentions, and behaviours and typically addressing attitudinal or knowledge-related barriers (Duthie *et al*. 2024). However, scarce research has examined campaign interventions with older men. A theory-informed exploration into the factors informing older men’s decisions about seeking help is necessary to guide the direction and design of more effective interventions promoting older men's help-seeking. Furthermore, non-metropolitan older men may face specific barriers to seeking help relative to their metropolitan (i.e. major city) counterparts, warranting research into whether help-seeking predictors differ among populations (Stewart *et al*. 2015). For this purpose, the reasoned action approach (RAA) helps understand help-seeking determinants to inform various health promotion interventions (Ajzen 1991, 2002, Fishbein and Cappella 2006).

The RAA has been successfully shown to predict mental health help-seeking intentions (Adams *et al*. 2022) and has informed numerous health interventions (Ajzen and Albarracín 2007). In the context of help-seeking, the theory proposes that older men form the intention to seek help, which is the most direct determinant of actual help-seeking behaviour. The RAA recognizes that intentions can translate into behaviour when an individual has the necessary skills and environmental factors facilitate rather than hinder the behaviour. Intention is a product of attitudes, perceived norms, and perceived behavioural control regarding help-seeking. Each construct comprises two distinct components: attitudes consists instrumental attitudes, which include beliefs about the practical consequences of a behaviour, and experiential attitudes, concerning the affective experience associated with a behaviour; perceived norms includes injunctive norms, which reflect whether a behaviour is socially approved or not by important others, and descriptive norms that refer to whether important others are engaging in a behaviour or not; lastly perceived behavioural control consists of perceived capacity, which is the perceived ability to engage in a behaviour, and perceived autonomy, involving the perceived decision-making capacity about whether or not to engage in a behaviour. These broader perceptions are underpinned by more specific behavioural beliefs (i.e. about consequences), normative beliefs (i.e. about social expectations), and control beliefs (i.e. about facilitators and barriers) (Ajzen 1991, 2002, Fishbein and Cappella 2006, Fishbein and Ajzen 2010). Concerning campaigns, the RAA posits a causal relationship whereby message-based interventions directly affect these specific help-seeking beliefs, shaping the broader perceptions of help-seeking as beneficial, socially acceptable, and easy to do. This process then strengthens intentions and increases the likelihood that older men will seek help for mental health (Fishbein and Cappella 2006). Such message-based interventions targeting health behaviours like physical activity have been shown to positively affect behaviours through these theorized routes (Wakefield *et al*. 2010). Distinguishing specific beliefs and broader perceptions about help-seeking is critical for designing effective messaging.

With the role of traditional masculinities and various attitudinal and system-level factors affecting men’s mental health service use (Yousaf *et al*. 2015), determining the influence of such factors on help-seeking is crucial. Research has also pointed to the need for more age-specific mental health promotion messaging (Malkin *et al*. 2019), as older adults have different informational processing styles relative to younger cohorts (Löckenhoff and Carstensen 2007). Since the RAA contends that belief change presupposes behavioural change, a critical first step is eliciting older men's most salient help-seeking beliefs to account for the influence of age and gender-related factors. Addressing these beliefs in any health promotion intervention would be a priority, as they would most likely influence help-seeking. Despite this, scarce research has adopted a theory-driven approach to examining older men’s beliefs influencing help-seeking for mental health concerns.

### Hypotheses and research questions

The study's primary aim was to inform the design of health promotion messaging to improve older men’s help-seeking for mental health. It drew on the work of Middlestadt *et al*. (1996) in translating the RAA into belief elicitation procedures and subsequent elaborations using mixed-methods approaches (Yzer *et al*. 2015). It involved a belief elicitation study (Study 1) to identify older men’s beliefs about help-seeking, and a closed-question survey (Study 2) that assessed which beliefs best explained behavioural intention to seek help. Study 2 also aimed to provide conceptual evidence for the RAA in explaining older men’s intention to seek help for mental health concerns and its utility in informing health promotion messaging. While this study does not predict actual help-seeking, the focus on intention is still warranted as it is the strongest determinant of behaviour, giving insights into help-seeking processes. Across this study, metropolitan participants included those living in major cities. All other participants were categorized as non-metropolitan due to sampling constraints, acknowledging that this may disregard contextual differences within this grouping. The study design aimed to answer the following research questions:

‘RQ1’ (Study 1): What do older men in metropolitan and non-metropolitan areas describe as the key beliefs around help-seeking for mental health concerns?
‘RQ2’ (Study 2): What are the associations between older men’s attitudes, perceived norms, and perceived behavioural control, and help-seeking intention, adjusting for significant demographic variables?
‘RQ3’ (Study 2): Which behavioural, normative, and control beliefs have the strongest influence on help-seeking intention, controlling for significant demographic variables?
‘RQ4’ (Study 2): Do the associations between beliefs, attitudes, perceptions, and help-seeking intention vary between older men in metropolitan and non-metropolitan areas?

## STUDY 1: BELIEF ELICITATION INTERVIEWS

### Methods

#### Procedures and participants

Participants had to be male and aged 65 years and older to partake in Study 1. Participants were recruited from the general public through convenience and snowball sampling through email lists of several organizations (e.g. Men’s Sheds and churches) across rural and metropolitan Australia. Twenty-three men aged between 65 and 89 (*M* age = 74.91, SD = 7.14) provided informed consent before participating in the interviews. Full sample characteristics are outlined in the Supplementary Table S1. While men were asked to report their current geographic location, several indicated having lived in both city and rural areas. Interviews were conducted via Zoom or in person based on participant location and preferences. Interviews were transcribed verbatim from recorded audio and checked for accuracy by one author (G.D.). Transcripts were de-identified before data analysis.

#### Instrument

The behaviour of interest was first specified, including a description of mental health professionals, including psychologists, psychiatrists, general practitioners, clinical social workers, and counsellors and mental health concerns, which could include depression, anxiety or suicidal thoughts. Participants were asked to imagine they experienced a mental health concern and to report on their views about making an appointment with a mental health professional in the next two months. Open-ended interview questions were asked about participants’ behavioural, normative, and control beliefs based on established approaches (Middlestadt *et al*. 1996, Yzer and Gilasevitch 2019). A single question was asked for each belief, which has been truncated for brevity. For behavioural beliefs, participants responded to: ‘What good/bad things might happen if you were to make an appointment with a mental health professional anytime in the next two months?’ and ‘What good or positive/negative things come to mind when you think about seeking help for mental health concerns?’ To examine normative beliefs, participants were asked for injunctive norms: ‘Are there any groups or people in your life who would approve/disapprove if you would make an appointment with a mental health professional anytime in the next two months?’ and for descriptive norms: ‘What individuals or groups of people do you think are most likely/unlikely to seek help for mental health concerns?’ Control beliefs were explored by asking: ‘What factors or circumstances would make it easier/difficult or impossible for you to make an appointment with a mental health professional anytime in the next two months?’

Demographic data collected included participants’ experience with mental health adapted from the Personal Experience to Mental Illness (PETMI) questionnaire (King *et al*. 2022) using a dichotomous (‘Yes’ or ‘No’) scale, age, highest education level, language spoken at home, location (i.e. major city with a population of 100 000 or more, regional—a small city or town outside a major city, rural—an area a few hours’ drive from a regional area, or remote—very far from a regional area), marital status, and employment status.

#### Analysis

Interview transcripts were coded using NVivo. One author (G.D.) conducted coding, including identifying text labels. Analysis was guided by the RAA, with responses categorized as behavioural, normative, or control beliefs. Responses were grouped into belief themes according to their conceptual similarity and topic relevance. Different ideas were separated to ensure that beliefs were mutually exclusive but representative. Coding was reviewed by another author (H.X.), and revisions were made until a consensus was reached. The raw data were then compared to the belief themes to ensure accuracy, with further refinements made accordingly by all authors (G.D., N.R., A.M., and H.X.). Lastly, beliefs were subjected to a frequency analysis to rank order and identify their relative salience within their category.

### Results

The 23 participants gave 601 belief references, representing 18 behavioural beliefs (i.e. consequences), 20 normative belief groups separated into injunctive and descriptive normative referents, and 14 control beliefs (i.e. facilitators and barriers) presented in Table 1. All belief groups were examined in Study 2, except for two normative referents: the injunctive norm of ‘No one’ and the descriptive norm of ‘People with mental health problems’ were removed. The group of ‘No one’ does not represent any specific belief system, making comparative analysis less meaningful. Additionally, ‘People with mental health problems’ potentially overlaps with all descriptive norm groups, obscuring the distinctions between the belief groups and associations with help-seeking intention.

**Table 1.** Description and frequency of beliefs about seeking help for mental health.

Behavioural beliefs	*n*	%	Normative beliefs	*n*	%	Control beliefs	*n*	%
**Positive**			**Approve**			**Facilitators**		
Begin to resolve my MH concerns or feel some relief	12	52	My family and significant others	17	74	Getting an appointment quickly	14	61
Have an understanding person listen to my MH concerns Obtain helpful new advice or strategies for my MH concerns	10 10	43 43	People in my friend and social circles	11	48	People encouraging help-seeking or talking openly about MH Professional recommendation to see another MH professional	13 12	57 52
Gain more understanding about myself and my MH concerns Receive reliable and confidential support from a professional Start to cope better or return to familiar activities Start to be more social, improve relationships, or help others	9 5 3 3	39 22 13 13	**Disapprove** No one People in my friend and social circles My family and significant others	15 4 2	65 17 8	Getting help in a way you preferred Affordable treatment or having financial means to pay for it Self-recognition of the need for help Someone in your life recognized you needed help	9 7 6 5	39 30 26 22
**Negative**			**Likely to seek help**			**Barriers**		
Feel disapproved or judged for having MH concerns	9	39	People with difficult or stressful life circumstances or events	8	35	Long wait times or limited services	13	57
Have to see a professional I do not get along with	9	39	People who recognize the value or need to seek help	5	22	Getting to appointments	7	30
Experience worse MH	5	22	Younger people in general	4	17	Unsure where to seek help or what help is available	7	30
Feel fearful about getting a difficult diagnosis	5	22	People with mental health problems	4	17	Cost of treatment	4	17
Discomfort with discussing things that cause me distress	5	22	People who are lonely	3	13	Health issues or incapacity	4	17
Feeling challenged in my values or mindset	3	13	Women in general	3	13	Not knowing or thinking you needed help	3	13
Feeling fearful about poor treatment outcomes	3	13	People with physical health problems	2	9	Busyness or lack of time	1	4
Discomfort with admitting weakness or needing help	3	13	People with good social support	2	9			
Experiencing medication side-effects	2	9	People with good life circumstances or events	2	9			
Discomfort with having to start making lifestyle changes	2	9	People with religious beliefs	2	9			
Having to start taking medication	2	9	Older people in general	1	4			
			People who know others who have sought help	1	4			
			People who know where to seek help	1	4			
			**Unlikely to seek help**					
			People who are self-reliant or stubborn in seeking help	8	35			
			People fearful of seeking help or showing weakness	6	26			
			Men in general	5	22			
			People with difficult or stressful life circumstances or events	3	13			
			People with good social support	3	13			
			Older people in general	3	13			
			People with mental health problems	3	13			
			Younger people in general	2	9			
			People whose professions are stereotyped as tough or stoic	2	9			
			People who are lonely	1	4			
			People with good life circumstances or events	1	4			

MH, mental health; *n*, number of people in the total sample who reported this belief; %, percentage of the total sample who reported this belief.

### Study 1 discussion

A range of help-seeking beliefs for mental health was elicited, leading to several important beliefs. Regarding important behavioural beliefs, positive consequences included improved mental health, new solutions, self-understanding, and being listened to. Negative consequences emerged around stigma and not getting along with the professional. Two injunctive referent groups were mentioned: family and significant others, and friends and social circles. Most participants (74%) indicated family approval of help-seeking, and no one would disapprove of help-seeking (65%). Those with negative life circumstances or who recognized the importance of help-seeking were reported as the most likely to seek help. Meanwhile, people perceived as self-reliant, stubborn, or fearful of seeking help or showing weakness, and men, were reported as being the least likely to seek help. Prominent control beliefs included available treatment, monetary factors, encouragement from others, recognition of needing help, and referral pathways. With these beliefs identified, Study 2 examined their association with intention within the RAA framework.

## STUDY 2: SURVEY

### Methods

#### Procedures and participants

Study 2 comprised a cross-sectional sample of men aged 65 or older in Australia. An Australian social research company, the Online Research Unit (ORU), was engaged to recruit male participants and circulate the survey. Participants were recruited from various channels, mostly offline (e.g. post and telephone), thus minimizing sampling biases. ORU uses stratified sampling to ensure a nationally representative sample reflective of the Australian population based on ABS data, including state location and age. All participants provided informed consent before partaking in the anonymous online survey hosted via Qualtrics. Three screening questions on age, gender, and Australian residency assessed eligibility. Eligible participants (*N* = 354) were automatically taken to the start of the survey, which took about 10 min to complete. An attention check question was included to manage response reliability, with 38 responses removed that failed or did not complete the check. Ten additional respondents dropped out early in the survey and were removed from the analyses. ORU compensated respondents for completing the survey. The final sample for analysis comprised 306 men aged between 65 and 95 (*M* age = 75.28, SD = 5.65), which was considered sufficient to detect medium effect sizes with adequate power (≥ 0.80). Full sample characteristics are outlined in Supplementary Table S1.

#### Measures

RAA variables were defined and measured in line with the theory and measurement approaches (Fishbein and Ajzen 2010). The target behaviour was phrased as ‘seeking help to discuss your mental health concerns with a mental health professional in the next two months,’ with the definition of mental health concerns and mental health professionals equivalent to Study 1. All questions asked participants to respond by imagining that they had a mental health concern, except for demographic and descriptive norm questions.

‘Intention to seek help’ was measured by asking participants, ‘How likely is it that you will make an appointment to see a mental health professional in the next 2 months?’ (1 = extremely unlikely, 7 = extremely likely). Concerning ‘attitudes’, six seven-point semantic differential items measured instrumental and experiential attitudes. Participants responded to the stem ‘Seeking help from a mental health professional in the next 2 months would be…’ with the following items: ‘extremely bad—extremely good’, ‘extremely harmful—extremely beneficial’, ‘extremely unnecessary—extremely necessary’, and ‘extremely useless—extremely useful’ (for instrumental attitude), and ‘extremely unpleasant—extremely pleasant’ and ‘extremely unsatisfying—extremely satisfying’ (for experiential attitude). Item scores were averaged to create a scale of instrumental attitude (Cronbach’s *α* = 0.91) and experiential attitude (Spearman-Brown coefficient *r*_SB_ = 0.83). ‘Perceived behavioural control’ was assessed with four seven-point items. For perceived capacity, participants were asked, ‘There can be obstacles to making an appointment. Even in the face of such obstacles, how sure are you that you could make an appointment?’ (1 = extremely unsure, 7 = extremely sure) and ‘How confident are you that you could make an appointment?’ (1 = not at all confident, 7 = completely confident). Two semantic differential items assessed perceived autonomy beginning with the stem ‘Making an appointment would be…’ followed by the items ‘not under my control—very much under my control’ and ‘not up to me—very much up to me’. An average score was then calculated for perceived capacity (*r*_SB_ = 0.93) and perceived autonomy (*r*_SB_ = 0.75). Two sets of items assessed ‘perceived norms’, separated into injunctive and descriptive norms. To measure injunctive norms, the stem ‘How would the following people or groups view your seeking help?’ was followed by ‘My family and significant others would…’ and ‘People in my friend and social circles would…’ with scale anchors of 1 (‘strongly disapprove’) to 7 (‘strongly approve’). Averaged scores formed an injunctive norms scale (*r*_SB_ = 0.83). Participants responded to the following question for descriptive norms: ‘How many of the people close to you (e.g. a family member or friend) do you think would seek help from a mental health professional if they had a mental health concern?’ (1 = almost none, 7 = almost all).

The selected Study 1 beliefs were converted into survey items. For the ‘behavioural beliefs’, the stem ‘How likely is it that the following will happen to you if you make an appointment? I would…’ was followed by each of the behavioural beliefs, with the response scale 1 (‘extremely unlikely’) to 7 (‘extremely likely’). The ‘control beliefs’ were measured with separate questions for the facilitators and barriers, truncated for brevity: ‘To what extent would the following assist/stop you in seeking help?’. Questions were followed by the control beliefs with response options 1 (‘not at all’) to 7 (‘very much’). ‘Normative beliefs’ were separated into injunctive and descriptive norms to better understand the influence of different referent groups. Injunctive norms were captured by disaggregating the injunctive norms scale into item scores for ‘My family and significant others’ and ‘People in my friend and social circles’. To measure descriptive norms, participants were asked, ‘In your opinion, how likely are the following individuals or groups to seek help for mental health concerns?’ The question was followed by the descriptive norm groups and the response scale, 1 (‘extremely unlikely’) to 7 (‘extremely likely’). Belief measures were not scaled as each belief was analysed separately.

Demographic questions were equivalent to Study 1, with two additional questions. First, the Kessler K6 (Kessler *et al*. 2002) was included to measure psychological distress, given its likely influence on help-seeking intention. The K6 consists of six items (e.g. ‘During the past 30 days, about how often did you feel nervous?’) with response options ranging from 0 (‘none of the time’) to 4 (‘all of the time’). The items were summed to determine the sample’s overall level of distress (*M* = 2.20, SD = 3.05, range = 0–20, Cronbach’s *α* = 0.82). Second, a dichotomous (‘yes’ or ‘no’) question asked participants whether they had ever sought help for mental health concerns from a mental health professional, with 23.53% of participants (*n* = 72) reporting prior help-seeking for mental health (one not reported).

#### Analysis

Analysis was conducted in SPSS, and descriptive analyses were performed to explore data distribution patterns and determine statistical approaches. Demographic variables significantly associated with help-seeking intention were analysed with bivariate correlations or linear regression. Demographic variables comprising more than two nominal categories were recoded into binary categories (e.g. retired or not). Concerning RQ2, bivariate correlations assessed associations between attitudes, perceived norms, perceived behavioural control, and help-seeking intention, which was re-run with linear regression, controlling for significant demographic variables. For RQ3, linear regression analysis tested the relationship between behavioural, normative, and control beliefs and intention, adjusting for significant demographic variables. Last, RQ4 assessed location-based effects by re-running linear regression analyses including an interaction term of belief/perception × location (i.e. metropolitan or non-metropolitan). Effect sizes of standardized beta values and Pearson’s *r* were interpreted as small (0.1), medium (0.3), or large effects (≥0.5).

### Results

On examining the influence of the demographic variables on help-seeking intention, only five were significantly associated with help-seeking intention: previous help-seeking (*r* = 0.24, 95% CI [0.13, 0.34], *P* < .001), location (i.e. metropolitan or non-metropolitan; *r* = −0.14, 95% CI [−0.25, −0.03], *P* = .013), language spoken at home (i.e. English or other; *r* = −0.12, 95% CI [−0.23, −0.01], *P* = .036) historical or current personal mental health concerns (*r* = 0.21, 95% CI [0.10, 0.31], *P* < .001), and current relationship with someone with a mental health concern (*r* = 0.14, 95% CI [0.03, 0.25], *P* = .016). The other demographic variables did not have a statistically significant relationship with intention (*P*’s > .05; see Supplementary Table S2). Thus, only these five variables were included in all subsequent multivariable analyses.

Table 2 shows that more favourable attitudes, perceived norms, and perceived behavioural control are positively associated with help-seeking intention. However, there was considerable variation in associations among variables. Only instrumental attitudes were strongly correlated with help-seeking intention (*r* = 0.64, 95% CI [0.57, 0.70], *P* < .001). Instrumental attitudes (*M* = 5.18) were more favourable than experiential attitudes (*M* = 4.48). Participants reported that people close to them (i.e. family or close friends) would likely approve of their help-seeking (*M* = 5.50) but that people close to them would be less likely themselves to seek help (*M* = 4.50). Participants felt quite autonomous (*M* = 5.71) and confident (*M* = 5.03) in seeking help, but were only just above the midpoint in their willingness to seek help (*M* = 4.65). Exploratory linear regression analyses by location found that non-metropolitan-based participants were significantly more reluctant to seek help (*M* = 4.29) than their metropolitan counterparts (*M* = 4.83; *b* = −0.54, 95% CI [−0.96, −0.12], *P* = .013, *β* = −0.14). Compared to metropolitan-based participants (*M* = 5.20), those living in non-metropolitan areas had a significantly lower confidence in seeking help (*M* = 4.70; *b* = −0.50, 95% CI [−0.84, −0.15], *P* = .005, *β* = −0.16). None of the other differences were significant by location (*P*’s > .05).

**Table 2.** Means (standard deviations) and bivariate correlations among reasoned action variables.

	Correlations (Pearson’s *r*)						Metropolitan		Non-metropolitan		Overall	
	IA	EA	PC	PA	IN	DN	*M*	SD	*M*	SD	*M*	SD
Help-seeking intention (HSI)	0.64***	0.45***	0.47***	0.16**	0.38***	0.43***	4.83	1.67	4.29	1.92	4.65	1.77
Instrumental attitudes (IA)	—	0.70***	0.53***	0.29***	0.48***	0.45***	5.25	1.16	5.03	1.28	5.18	1.20
Experiential attitudes (EA)		—	0.44***	0.26***	0.38***	0.37***	4.49	1.00	4.46	1.41	4.48	1.15
Perceived capacity (PC)			—	0.52***	0.39***	0.33***	5.20	1.26	4.70	1.76	5.03	1.46
Perceived autonomy (PA)				—	0.25***	0.16**	5.74	1.01	5.64	1.29	5.71	1.11
Injunctive norms (IN)					—	0.53***	5.52	1.04	5.46	1.14	5.50	1.08
Descriptive norms (DN)						—	4.59	1.53	4.32	1.81	4.50	1.63

**P ≤ .01, ***P ≤ .001.

Multivariable regression analyses for RQ2 found that when controlling for significant demographic variables, all RAA variables remained significant predictors of intention (see Table 3). Among them, instrumental attitudes had the strongest association with intention (*b* = 0.88, 95% CI [0.75, 1.01], *P* < .001, *β* = 0.60), followed by perceived capacity (*b* = 0.53, 95% CI [0.41, 0.65], *P* < .001, *β* = 0.44), experiential attitudes (*b* = 0.67, 95% CI [0.52, 0.82], *P* < .001, *β* = 0.43), and descriptive norms (*b* = 0.43, 95% CI [0.32, 0.54], *P* < .001, *β* = 0.40).

**Table 3.** Reasoned action variables regressed with intention.

	Variables				Model
	*b*	SE *b*	*β*	*P* value	*R* ^2^
Instrumental attitudes	0.88	0.07	0.60	<.001	0.45
Experiential attitudes	0.67	0.08	0.43	<.001	0.29
Perceived capacity	0.53	0.06	0.44	<.001	0.29
Perceived autonomy	0.20	0.09	0.13	.023	0.12
Injunctive norms	0.57	0.09	0.34	<.001	0.22
Descriptive norms	0.43	0.06	0.40	<.001	0.26

Models adjusted for location, language spoken at home, previous help-seeking behaviour, current or historical personal mental health concerns, and current relationship to someone with mental health concerns.

Table 4 shows that a diverse set of behavioural, normative, and control beliefs shapes help-seeking intention among participants (RQ3). Only medium and above effect sizes (i.e. *β* ≥ 0.30) were considered salient. Specifically, influential behavioural beliefs were primarily about the positive outcomes of seeking help, including receiving reliable and confidential support (*b* = 0.59, 95% CI [0.46, 0.72], *P* < .001, *β* = 0.44), improved self-understanding (*b* = 0.59, 95% CI [0.45, 0.72], *P* < .001, *β* = 0.43), obtaining helpful strategies or advice (*b* = 0.57, 95% CI [0.44, 0.70], *P* < .001, *β* = 0.42), being listened to (*b* = 0.57, 95% CI [0.44, 0.70], *P* < .001, *β* = 0.42), starting to cope better or resume familiar activities (*b* = 0.59, 95% CI [0.44, 0.74], *P* < .001, *β* = 0.39), improved social engagement (*b* = 0.51, 95% CI [0.37, 0.65], *P* < .001, *β* = 0.38), and improved mental health outcomes (*b* = 0.52, 95% CI [0.37, 0.66], *P* < .001, *β* = 0.35). Intention was associated with likely approval from all injunctive referent groups, but principally family and significant others (*b* = 0.54, 95% CI [0.38, 0.70], *P* < .001, *β* = 0.34). Intention was further associated with several influential descriptive referent groups including older adults (*b* = 0.47, 95% CI [0.33, 0.61], *P* < .001, *β* = 0.35), people with good life circumstances (*b* = 0.40, 95% CI [0.27, 0.53], *P* < .001, *β* = 0.33), people who know others who have previously sought help (*b* = 0.55, 95% CI [0.37, 0.73], *P* < .001, *β* = 0.32), and people who know where to seek help (*b* = 0.57, 95% CI [0.38, 0.76], *P* < .001, *β* = 0.31). Help-seeking intention was mainly influenced by facilitating control beliefs such as encouragement from others (*b* = 0.51, 95% CI [0.39, 0.63], *P* < .001, *β* = 0.43), clear referral pathways (*b* = 0.52, 95% CI [0.39, 0.65], *P* < .001, *β* = 0.41), getting an appointment quickly (*b* = 0.47, 95% CI [0.35, 0.59], *P* < .001, *β* = 0.41), someone close recognizing distress (*b* = 0.47, 95% CI [0.35, 0.59], *P* < .001, *β* = 0.39), and personal recognition of needing help (*b* = 0.53, 95% CI [0.38, 0.67], *P* < .001, *β* = 0.38).

**Table 4.** Means (standard deviations) for behavioural, normative, and control beliefs regressed with intention.

	Metropolitan		Non-metropolitan		Overall		Belief				Model
	*M*	SD	*M*	SD	*M*	SD	*b*	SE *b*	*β*	*P* value	*R* ^2^
**Behavioural beliefs**											
Receive reliable and confidential support from a professional	5.33	1.22	5.22	1.52	5.30	1.32	0.59	0.07	0.44	<.001	0.30
Gain more understanding about myself and my MH concerns	5.30	1.20	5.20	1.52	5.26	1.31	0.59	0.07	0.43	<.001	0.30
Obtain helpful new advice or strategies for my MH concerns	5.12	1.23	4.99	1.49	5.08	1.32	0.57	0.07	0.42	<.001	0.29
Have an understanding person listen to my MH concerns	5.39	1.24	5.30	1.42	5.36	1.30	0.57	0.07	0.42	<.001	0.28
Start to cope better or return to familiar activities	4.98	1.11	5.04	1.31	5.00	1.18	0.59	0.08	0.39	<.001	0.26
Start to be more social, improve relationships, or help others	4.75	1.23	4.46	1.41	4.65	1.30	0.51	0.07	0.38	<.001	0.25
Begin to resolve my MH concerns or feel some relief	4.93	1.10	4.95	1.43	4.93	1.22	0.52	0.08	0.35	<.001	0.23
Discomfort with discussing things that cause me distress	3.82	1.63	3.54	1.88	3.73	1.72	−0.28	0.06	−0.27	<.001	0.18
Feel disapproved or judged for having MH concerns	3.61	1.52	3.54	1.80	3.59	1.61	−0.28	0.06	−0.25	<.001	0.18
Discomfort with admitting weakness or needing help	3.86	1.67	3.49	1.92	3.74	1.76	−0.25	0.06	−0.25	<.001	0.17
Have to see a professional I do not get along with	3.60	1.37	3.83	1.76	3.67	1.51	−0.27	0.06	−0.23	<.001	0.17
Feeling challenged in my values or mindset	3.85	1.38	3.66	1.68	3.79	1.49	−0.26	0.07	−0.22	<.001	0.16
Discomfort with having to start making lifestyle changes	4.34	1.44	4.04	1.78	4.24	1.56	−0.17	0.06	−0.15	.009	0.13
Experience worse MH	2.91	1.33	3.01	1.49	2.94	1.38	−0.15	0.07	−0.12	.031	0.13
Feeling fearful about poor treatment outcomes	3.84	1.45	3.81	1.66	3.83	1.52	−0.12	0.06	−0.10	.064	0.12
Having to start taking medication	4.27	1.46	4.20	1.59	4.25	1.50	0.08	0.07	0.06	.255	0.12
Experiencing medication side-effects	3.53	1.40	3.51	1.54	3.53	1.44	−0.06	0.07	−0.05	.359	0.12
Feel fearful about getting a difficult diagnosis	3.96	1.58	3.82	1.68	3.92	1.61	−0.03	0.06	−0.03	.647	0.11
**Normative beliefs**											
**Injunctive norm groups**											
My family and significant others	5.80	1.08	5.73	1.21	5.77	1.12	0.54	0.08	0.34	<.001	0.22
People in my friend and social circles	5.25	1.20	5.19	1.24	5.23	1.21	0.44	0.08	0.29	<.001	0.20
**Descriptive norm groups**											
Older people in general	3.99	1.24	4.00	1.46	3.99	1.31	0.47	0.07	0.35	<.001	0.23
People with good life circumstances or events	4.67	1.35	4.22	1.55	4.53	1.43	0.40	0.07	0.33	<.001	0.21
People who know others who have sought help	5.53	1.00	5.33	1.11	5.46	1.04	0.55	0.09	0.32	<.001	0.21
People who know where to seek help	5.63	0.89	5.48	1.14	5.58	0.98	0.57	0.10	0.31	<.001	0.21
People with good social support	5.07	1.18	4.98	1.40	5.04	1.26	0.37	0.08	0.27	<.001	0.18
Women in general	4.97	0.91	4.82	1.23	4.92	1.03	0.36	0.10	0.20	<.001	0.15
People who are self-reliant or stubborn in seeking help	2.67	1.22	2.44	1.32	2.59	1.26	0.26	0.08	0.19	<.001	0.15
People with physical health problems	4.15	1.13	3.90	1.33	4.07	1.20	0.27	0.08	0.18	<.001	0.15
Men in general	3.29	1.14	3.12	1.18	3.23	1.16	0.28	0.09	0.18	.001	0.14
People who recognize the value or need to seek help	5.70	1.01	5.63	1.13	5.68	1.05	0.31	0.10	0.18	.001	0.14
People fearful of seeking help or showing weakness	2.97	1.17	2.99	1.51	2.97	1.29	0.24	0.07	0.17	.002	0.14
People whose professions are stereotyped as tough or stoic	3.37	1.25	3.00	1.41	3.25	1.31	0.22	0.07	0.16	.004	0.14
People with religious beliefs	3.78	1.22	3.48	1.45	3.68	1.30	0.14	0.08	0.10	.070	0.12
Younger people in general	3.81	1.47	3.62	1.56	3.75	1.50	−0.05	0.07	−0.04	.455	0.11
People who are lonely	3.78	1.44	3.66	1.62	3.74	1.50	0.01	0.07	0.01	.876	0.11
People with difficult or stressful life circumstances or events	4.01	1.42	3.97	1.51	4.00	1.45	−0.01	0.07	−0.01	.923	0.11
**Control beliefs**											
People encouraging help-seeking or talking openly about MH	5.09	1.48	4.81	1.52	5.00	1.50	0.51	0.06	0.43	<.001	0.29
Professional recommendation to see another MH professional	5.59	1.35	5.56	1.49	5.58	1.40	0.52	0.06	0.41	<.001	0.27
Getting an appointment quickly	5.36	1.41	4.97	1.73	5.23	1.53	0.47	0.06	0.41	<.001	0.27
Someone in your life recognized you needed help	5.31	1.46	5.14	1.51	5.25	1.47	0.47	0.06	0.39	<.001	0.26
Self-recognition of the need for help	5.63	1.24	5.56	1.40	5.61	1.29	0.53	0.07	0.38	<.001	0.25
Getting help in a way you preferred	5.21	1.34	5.06	1.57	5.16	1.42	0.37	0.07	0.29	<.001	0.19
Cost of treatment	4.31	1.99	4.87	1.94	4.49	1.99	−0.23	0.05	−0.25	<.001	0.18
Getting to appointments	2.95	1.67	3.12	1.89	3.01	1.74	−0.25	0.05	−0.25	<.001	0.17
Unsure where to seek help or what help is available	3.73	1.65	3.72	1.76	3.73	1.68	−0.18	0.06	−0.17	.003	0.14
Not knowing or thinking you needed help	4.50	1.61	4.16	1.76	4.39	1.66	−0.17	0.06	−0.16	.003	0.14
Long wait times or limited services	4.14	1.76	4.23	1.86	4.17	1.79	−0.15	0.05	−0.15	.006	0.14
Busyness or lack of time	2.60	1.54	2.65	1.59	2.61	1.55	−0.17	0.06	−0.15	.006	0.14
Health issues or incapacity	3.49	1.66	3.34	1.71	3.44	1.68	−0.16	0.06	−0.15	.007	0.13
Affordable treatment or having financial means to pay for it	5.33	1.53	5.37	1.54	5.34	1.53	0.17	0.06	0.15	.007	0.13

Models adjusted for location, language spoken at home, previous help-seeking behaviour, current or historical personal mental health concerns, and current relationship to someone with mental health concerns.

Analyses on the impact of location (RQ4) found that when including an interaction term of belief/variable × location (i.e. metropolitan or non-metropolitan) for the RAA variables, none of the interaction terms were statistically significant (all *P*’s > 0.05). When examining the interaction term for the behavioural, normative, and control beliefs, only two control beliefs had statistically significant results. The cost of treatment interaction term was significant (*b* = −0.23, 95% CI [−0.43, −0.02], *P* = .032, *β* = −0.48) but the belief was non-significant (*b* = 0.07, 95% CI [−0.22, 0.35], *P* = .645, *β* = 0.07; model R^2^ = 0.19). Simple slopes analysis showed that the cost of treatment was a significant predictor of help-seeking in both location groups. Specifically, in the non-metropolitan group, the cost of treatment significantly predicted help-seeking (*b* = −0.40, 95% CI [−0.58, −0.22], *P* < .001, *β* = −0.40), indicating a stronger negative relationship. In the metropolitan group, the effect was smaller but still significant (*b* = −0.16, 95% CI [−0.27, −0.05], *P* = .006, *β* = −0.19). The interaction term for the belief around not knowing or thinking help is needed was significant (*b* = 0.33, 95% CI [0.09, 0.57], *P* = .006, *β* = 0.55) and the belief remained significant (*b* = −0.63, 95% CI [−0.97, −0.28], *P* < .001, *β* = −0.58; model R^2^ = 0.16). In the metropolitan group, the belief was a significant predictor of help-seeking (*b* = −0.30, 95% CI [−0.43, −0.17], *P* < .001, *β* = −0.29), revealing a small negative relationship. By contrast, in the non-metropolitan group, the effect was small and non-significant (*b* = 0.04, 95% CI [−0.17, 0.26], *P* = .704, *β* = 0.04). All other interaction terms within the models for all three beliefs were non-significant (*P*’s > .05).

### Study 2 discussion

The significant associations between all RAA variables provided helpful conceptual support for the RAA, with the perceptions also having predictive utility for participants’ intention to seek help, particularly instrumental attitudes. The analyses provided insight to inform health promotion interventions with older men, revealing that 84.0% (42/50) of the beliefs were significant determinants of help-seeking intention. Of these, 40.5% (17/42) represented salient (i.e. *β* ≥ 0.30) beliefs underpinning help-seeking intention. However, none of these salient beliefs significantly varied between location groups. Addressing these salient beliefs as part of an age-specific mental health promotion intervention could theoretically translate into the largest changes in help-seeking intention.

## DISCUSSION

With growing interest in health promotion activities aimed at improving older men’s use of mental health services, this study used a theory-driven approach to elicit older men’s most salient beliefs influencing intention to seek help for mental health concerns to primarily inform campaign messaging. The study also examined whether messaging might need to differ between metropolitan and non-metropolitan locations. The findings also highlight that a multipronged approach is necessary to address attitudinal, knowledge, and structural-related beliefs, whereby campaigns addressing several salient beliefs should be complemented by efforts to make mental health services more accessible for older men. To the authors’ knowledge, this is the first test of the RAA and mixed-methods approach to explain older men’s help-seeking intention for mental health concerns.

Applying the RAA through qualitative interviews produced 50 unique behavioural, normative, and control beliefs subjected to a closed-question survey, most of which constituted significant determinants of help-seeking intention. Having many specific salient beliefs that older men hold around help-seeking for mental health greatly enriches the understanding of their help-seeking process. Nonetheless, with the reported help-seeking intention slightly above the midpoint, more health promotion interventions targeting older men are needed. Accordingly, the salient beliefs identified are strong candidates for directly addressing in mental health promotion messaging with older men, including encouragement from others, gaining new solutions, improved self-understanding, and being listened to and supported in treatment, which should be coupled with system reforms.

Behavioural beliefs about the consequences of seeking help also appeared to influence older men’s help-seeking intention. As help-seeking requires action in the short term, older men found beliefs that emphasized more tangible or immediate benefits as more salient (e.g. returning to familiar activities) than longer-term or negative consequences (e.g. poor treatment outcomes). This aligns with research highlighting older adults’ preference for more present-oriented goals and positively framed information (Carstensen *et al*. 1999, Carstensen *et al*. 2003, Reed *et al*. 2014). This may be one reason why the negative consequences of stigma and admitting weakness from seeking help were perceived as less salient, despite their incompatibility with older men’s masculinity and research showing stigma and perceived weakness discouraging men’s help-seeking across the lifespan (Lynch *et al*. 2016, Thompson and Langendoerfer 2016, McKenzie *et al*. 2022). Influential care-related beliefs included receiving reliable and confidential support and being listened to, which were rated among the highest likelihood to occur, in line with research that men across the lifespan prefer to speak about their concerns in an understanding and safe healthcare environment that minimizes stigma, demonstrates empathy, and is sensitive to their perspective (Smith *et al*. 2008, Cheshire *et al*. 2016). Campaign messaging could capture such beliefs, which has scarcely been done to date. This should be coupled with health system changes to ensure messaging matches actual service experiences. For example, tailoring services to affirm supportive, safe, and non-judgemental environments for older men, such as with peer support programmes, while equipping mental health professionals to adopt male-centric and empathetic communication and treatment approaches that build trust, rapport, and effectively engage older men (Smith *et al*. 2008, Rice *et al*. 2020, Staiger *et al*. 2020). Interestingly, older men rated experiencing worse mental health as the lowest likelihood of occurring, suggesting favourable attitudes towards service outcomes consistent with research about older adults’ positive treatment beliefs (Mackenzie *et al*. 2008). Other salient beliefs included improving mental health, gaining self-understanding, improving social engagement, obtaining helpful strategies or advice, gaining coping skills, and returning to familiar activities. Although some of these help-seeking benefits have been utilized in campaigns such as ‘I found support that worked’ (Suicide Prevention Australia 2023), these findings highlight the need for more campaign messaging coupled with system reforms targeting short-term and noticeable benefits to strengthen older men’s behavioural beliefs around help-seeking for mental health.

At the normative level, the results emphasized that the perceptions of others around seeking help for mental health concerns can play a vital role in shaping intention. Foremost, participants highlighted the importance of approval from family and significant others across both studies, which aligns with research showing that family can encourage men’s help-seeking (Fisher *et al*. 2024). The influence of friends on intention was significant but not salient. This aligns with research highlighting that as adults get older, they prioritize closer relationships to optimize their emotional wellbeing (Carstensen *et al*. 1999, 2003), suggesting that adults deprioritize the influence of friends with age. Thus, messaging may benefit from references to likely approval and support of family in older men’s help-seeking. Interestingly, there was alignment across both studies that the least likely to seek help included those who are self-reliant or stubborn, fearful of seeking help or showing weakness, and men in general. Both studies agreed that people who recognized the value or need to seek help were among the most likely groups to seek help. However, this was not salient. Instead, knowing that other older adults and people with good life circumstances are seeking help may create a normative incentive for help-seeking. Relatedly, knowing others who have sought help or know where to seek help may influence help-seeking through signalling social support. However, few campaigns have incorporated such normative influences, which could be effective, particularly if combined with male-sensitive language (e.g. humour, non-stigmatizing language) that associates masculine ideals such as strength with help-seeking (Robertson *et al*. 2018). Healthcare professionals could also focus more on normalzsing older men’s help-seeking and treatment experiences to reduce stigma when accessing services (Rice *et al*. 2020). Thus, campaign messaging and healthcare professionals emphasizing that other older adults seek help, particularly those respected in the community or perceived to have good life circumstances, may point to influential social role models reinforcing older men’s help-seeking intention.

In terms of salient control beliefs, these primarily involved social aspects of motivation and problem identification to drive help-seeking, stemming from need or distress recognition by oneself or others and encouragement by others to seek help or talk about the issue, consistent with other studies with older adults (Wuthrich and Frei 2015, Teo *et al*. 2022), although encouragement to seek help has mixed findings as a facilitator with men in general (Seidler *et al*. 2020, Fisher *et al*. 2024). The current result suggests that when older men are socially supported, they feel less isolated and stigmatized and feel understood and empowered, which may prompt help-seeking. This aligns with Addis and Mahalik (2003) in that normalizing help-seeking and sharing concerns in social support groups may facilitate men’s help-seeking. The importance of problem identification highlights that awareness-raising campaigns to improve mental health literacy, particularly among older men and their families, may aid in recognizing distress and what support is available. Such salient beliefs have been used in several campaigns encouraging open mental health conversations and educating men about symptoms (Movember 2019, British Association for Counselling and Psychotherapy 2023). Other salient beliefs of getting a referral and appointment quickly represent structural aspects of the healthcare system in ensuring older men with mental health concerns access the right services, at the right time, and in an efficient way, which have not been a focus of campaigns to date but align with research on system navigational barriers to seeking help for men in general (Rice *et al*. 2018, Seidler *et al*. 2019). Thus, campaign messaging could de-mystify services, informing older men about the value of speaking to a healthcare professional about accessing services, where and how to access various services available, and which may be faster to access. Messaging may struggle to gain traction without re-orienting systems to be accessible for older men, such as by streamlining and hastening referral processes and having available appointments, while ensuring older men are empowered with choice and ownership over their help-seeking process (Smith *et al*. 2007, 2008). Interestingly, affordable treatment or having the financial means was rated a strong facilitator but not a good predictor of intention, aligning with findings that although treatment cost is a key barrier, it does not predict help-seeking for men across the lifespan (Wuthrich and Frei 2015, Yousaf *et al*. 2015, Rice *et al*. 2020). Ultimately, messaging approaches alongside system changes that make help-seeking easy and requiring minimal effort in appearance or practice may be ideal for strengthening older men’s perceived behavioural control around help-seeking.

As proximal determinants of intention, the results showed that some salient attitudes and perceptions shaped older men’s help-seeking intention. In particular, intention was primarily explained by instrumental attitudes, perceived capacity, experiential attitudes, and descriptive norms. This aligns with research identifying important RAA variables in help-seeking intention across broad populations (Shi *et al*. 2024). However, the current results found more moderate to strong effect sizes and that experiential attitude was more influential than previous findings, which may relate to research indicating that older adults prefer more emotionally meaningful information (Carstensen *et al*. 1999, 2003). These results have practical implications, suggesting that older men may benefit from campaign messaging that addresses underlying beliefs associated with these perceptions. Specifically, messaging could focus on behavioural beliefs about positive outcomes or affective experiences about seeking help (e.g. there are many benefits to seeking help such as feeling some relief), control beliefs relating to building skills and confidence to seek help (e.g. talk to your doctor about how to access mental health services), and normative beliefs that pertain to what important others are seeking help (e.g. it is common for men your age to seek help for mental health concerns).

There were only a few differences between metropolitan and non-metropolitan participants, acknowledging that grouping regional, rural, and remote areas as non-metropolitan may overlook potential contextual nuances within these populations. Notably, non-metropolitan participants were more sensitive to cost as a barrier. Meanwhile, metropolitan participants were less likely to seek help if they did not know or think they needed it. Additionally, the belief about needing help did not appear to influence non-metropolitan participants’ intention. Although these findings suggest the need for location-specific campaign messaging targeting these beliefs, neither was considered salient. Thus, other salient beliefs that did not vary could be prioritized for health promotion efforts. Non-metropolitan-based participants had a significantly lower help-seeking intention and confidence, which may relate to higher rated control beliefs concerning barriers around cost and getting to appointments among non-metropolitan participants, also shown in other research among non-metropolitan populations (Keeves *et al*. 2021, Kavanagh *et al*. 2023). While not salient, health services should avoid reinforcing these beliefs by ensuring service accessibility and affordability outside metropolitan areas by subsidizing, enlisting, and retaining more rural mental health professionals (Kavanagh *et al*. 2023). Relatedly, non-metropolitan participants’ lower intention concurs with existing research (Kaukiainen and Kõlves 2020), highlighting the need for more campaigns targeting non-metropolitan-based older men.

### Limitations

Interpreting this study’s results may be hindered by several limitations. The findings may not represent all older men, which restricts discussion around their generalizability. While there was some variation in the participant characteristics, convenience sampling may have introduced some selection bias, such as the small number of participants who did not speak English at home. It is possible that some men chose not to participate because of the study’s design (e.g. being conducted in English, closed-question online survey), affecting external validity. As the recruitment materials disclosed the topic of mental health, participants with more positive attitudes towards mental health may have been more likely to participate, hindering the generalizability of findings. Response bias among certain participants (e.g. those with strong mental health views) may also be possible. Despite efforts to recruit additional non-metropolitan participants, unequal sample size groups in Study 2 may have reduced statistical power to detect differences between location groups. Although the mixed methods approach provided an extensive list of beliefs associated with intention, other factors or beliefs that strongly influence intention might not be captured.

### Future directions

Although campaign developers or health promotion practitioners should prioritize the identified salient belief targets, it remains unclear exactly how these beliefs could be translated into campaign messaging for older men. Thus, more translational research is needed examining older men’s perceptions of how these beliefs can be framed, delivered, and tested as campaign messaging, complemented by similar research for system-related findings. Prospective research could explore factors influencing whether older men act on their intention to seek help, as understanding the intention-behaviour gap may be necessary for designing more effective health promotion initiatives. Relatedly, although few location-based differences were noted in the salience of beliefs and variables on intention, effective campaign message tailoring may differ within non-metropolitan older men, and further exploration of unique remote, rural, and regional perspectives is warranted. Research would benefit from better defining optimal campaign segmentation strategies to improve help-seeking for mental health concerns among older men.

## CONCLUSION

The current findings highlight that the RAA provides a useful framework for understanding older men’s most salient beliefs influencing their intention to seek help for mental health concerns and guide health promotion interventions in metropolitan and non-metropolitan locations. Most RAA variables had good predictive power on help-seeking intention, which did not vary by location. The findings from this study offer direction for future health promotion campaigns by identifying influential candidates to address in messaging with older men, which should be supported by enhancements in health service accessibility for older men. Although metropolitan and non-metropolitan older men did not vary on salient beliefs, research is required to refine message design and delivery to promote help-seeking for mental health concerns among older men.

## Supplementary Material

daaf170_Supplementary_Data

## Data Availability

Data available within the article or its Supplementary materials.
