# Supplementary material for: Beliefs that explain mental health help-seeking intention among older men: a reasoned action approach
Source: Health Promot Int. 2025 Oct 6;40(5):daaf170. doi: 10.1093/heapro/daaf170 (PMC12497511; doi:10.1093/heapro/daaf170)
Supplement: daaf170_Supplementary_Data [file daaf170_supplementary_data.zip › Table S1.doc]

**Table S1**

*Demographic Variables From Studies 1 & 2*

| Variable | Study 1 | | Study 2 | |
| --- | --- | --- | --- | --- |
|  | *n* | % | *n* | % |
| Highest education level |  |  |  |  |
| Primary school | 1 | 4.35 | 2 | 0.65 |
| High school | 6 | 26.09 | 92 | 30.07 |
| TAFE/institution | 4 | 17.39 | 102 | 33.33 |
| University | 12 | 52.17 | 110 | 35.95 |
| Employment status* |  |  |  |  |
| Unemployed | 1 | 4.35 | 1 | 0.33 |
| Employed | 1 | 4.35 | 31 | 10.13 |
| Retired | 20 | 86.96 | 260 | 84.97 |
| Pension | 5 | 21.74 | 28 | 9.15 |
| Home duties | 1 | 4.35 |  |  |
| Location |  |  |  |  |
| Major city | 7 | 30.43 | 206 | 67.32 |
| Regional area | 10 | 43.48 | 80 | 26.14 |
| Rural area | 6 | 26.09 | 19 | 6.21 |
| Remote area |  |  | 1 | 0.33 |
| Marital status |  |  |  |  |
| Single (never married) | 3 | 8.70 | 20 | 6.54 |
| Widowed | 3 | 13.04 | 25 | 8.17 |
| Divorced | 2 | 13.04 | 39 | 12.75 |
| Married | 15 | 65.22 | 202 | 66.01 |
| De facto |  |  | 14 | 4.58 |
| Separated not divorced |  |  | 6 | 1.96 |
| Language spoken at home |  |  |  |  |
| English | 20 | 86.96 | 296 | 96.73 |
| German | 2 | 8.70 | 1 | 0.33 |
| French |  |  | 2 | 0.65 |
| Chinese (Cantonese or Mandarin) |  |  | 2 | 0.65 |
| Indonesian |  |  | 2 | 0.65 |
| Spanish |  |  | 1 | 0.33 |
| Italian |  |  | 1 | 0.33 |
| Not reported | 1 | 4.35 | 1 | 0.33 |
| Personal Exposure to Mental Illness** |  |  |  |  |
| Current or historical MI | 8 | 34.78 | 85 (3 NR) | 27.78 |
| Relationship to someone with MI | 4 | 17.39 | 16 (1 NR) | 5.23 |
| Has a friend with MI | 9 | 39.13 | 65 (1 NR) | 21.24 |
| Grew up around someone with MI | 7 | 30.43 | 25 (1 NR) | 8.17 |
| Degree of experience with mental health concerns** |  |  |  |  |
| 0 | 5 | 21.74 | 168 | 54.90 |
| 1 | 10 | 43.48 | 98 | 32.03 |
| 2 | 6 | 26.09 | 28 | 9.15 |
| 3 | 2 | 8.70 | 11 | 3.59 |
| 4 |  |  | 1 | 0.33 |

*Note*. * Participants could select multiple options that apply. **Indicates the sum of participants’ “Yes” responses to questions. MI = mental illness. NR = not reported.
