# Supplementary material for: Beliefs that explain mental health help-seeking intention among older men: a reasoned action approach
Source: Health Promot Int. 2025 Oct 6;40(5):daaf170. doi: 10.1093/heapro/daaf170 (PMC12497511; doi:10.1093/heapro/daaf170)
Supplement: daaf170_Supplementary_Data [file daaf170_supplementary_data.zip › Table S2.doc]

**Table S2**

Bivariate Correlations (Pearson’s r) for Demographic Variables With Intention

|  | Correlation (*r*) | *p* value |
| --- | --- | --- |
| Highest education level | .01 | .816 |
| Employment status | .01 | .851 |
| Location (i.e., metropolitan or non-metropolitan) | -.14 | .013 |
| Marital status | .11 | .060 |
| Language spoken at home | -.12 | .036 |
| Current or historical MI | .21 | <.001 |
| Relationship to someone with MI | .14 | .016 |
| Has a friend with MI | .10 | .082 |
| Grew up around someone with MI | .05 | .431 |
| K6 | .08 | .188 |
| Previous help-seeking | .24 | <.001 |
| Age | .01 | .902 |

*Note*. MI = mental illness.
